# Subgenomic sequence analysis reveals emergence of new circulating recombinant forms of HIV-1 in Pakistan

**DOI:** 10.12669/pjms.38.7.5591

**Published:** 2022

**Authors:** Maria Zahid, Saeed Khan, Muhammad Asif Qureshi, Yasir Raza

**Affiliations:** 1 Maria Zahid, M.Phil. Department of Molecular Pathology, Dow University of Health Sciences, Karachi – Pakistan, Department of Microbiology, University of Karachi, Karachi - Pakistan; 2 Saeed Khan, PhD. Department of Molecular Pathology, Dow University of Health Sciences, Karachi – Pakistan, Department of Pathology, Dow University of Health Sciences, Karachi - Pakistan; 3 Muhammad Asif Qureshi, PhD. Department of Pathology, Dow University of Health Sciences, Karachi - Pakistan; 4 Yasir Raza, PhD. Department of Microbiology, University of Karachi, Karachi - Pakistan

**Keywords:** HIV, Novel Mutations, Durg Resistance

## Abstract

**Background and Objective:**

Pakistan has witnessed a dramatic change in the increasing prevalence and emergence of HIV subtypes for more than two decades. Pakistani population is increasingly engaged in high-risk practices, and the prescribed drugs are potentially causing resistance. There are chances that these resistant strains are beginning to circulate from high-risk to the general population.

**Methods:**

The study was conducted at the section of Molecular Pathology Lab of Dow Diagnostic and Research Laboratory, Dow University of Health Sciences (DUHS) Karachi. In this study, we analyzed gene sequences of HIV for drug resistance and molecular epidemiology., along with amino acid sequence variability. Furthermore, we undertook phylogenetic analysis for possible geographic linkages of Pakistani HIV strains.

**Results:**

Our results demonstrate that A1 is the leading HIV subtype circulating in the country, whereas other emerging subtypes and recombinant forms, including subtype B, CRF02_AG, CRF10_CD CRF35_AD, and CRF11_cpx were also observed. Our sequences cluster with the Middle East, African, and a few European sequences according to geographical distribution. These sequences showed high-level resistance per drug resistance pattern, with 62.5% of patients exhibiting resistance to NNRTI drugs and 60% mutation at E138A and K103N, respectively, against NNRTI drugs. About 75% sequences showed resistance mutation at M184V against NRTI drugs. The antiretroviral drugs are now causing H-LR to the patients with no effect. Our results also revealed that certain regions of RT exhibited high sequence variability, especially at Amino Acids positions p.119, p.130, p.157, p.164.

**Conclusion:**

We hereby report major novel mutations and several minor mutations that may have a drastic change in the drug resistance pattern.

## INTRODUCTION

A total of 0.24 million people live with HIV in Pakistan, and 46,912 are registered with the Pakistan National AIDS control program (NACP). There has been a continuous increase in new cases since the first HIV case was identified.^1^ Previously it was reported that the HIV epidemic is considered to be in its early stage, but now a shift in the epidemic pattern has been observed from the last two decades.^2^

It was revealed from various phylogenetic analysis around the world that HIV-1 can be divided into three major groups M, N, and O, where M is majorly responsible for the global epidemic and is subdivided into A-K, more than 90 different circulating recombinant forms (CRFs) and several unique recombinant forms (URF).^3^,^4^ (http://www.hiv.lanl.gov/content/index)

It was observed that subtypes and CRFs have also emerged in the last few years. This distribution of HIV subtype across the globe is due to people involving in high-risk activities like Intravenous Drug Users (IDUs).^5^ It may also be due to error-prone activity of HIV that causes several mutations.

Antiretroviral therapy (ART) is used for the treatment of HIV-1 and can develop resistance due to mutations in HIV that can cause this therapy to become ineffective.^6^ Therefore, updated data regarding administered drugs is necessary to represent the resistance and sensitivity regiments against the virus.^7^

Therefore, in this study, we have analyzed the *pol* gene of HIV to explore the genetic diversity and pattern of drug resistance displayed by ART in Pakistan. We also evaluated the possible geographic linkages of HIV isolates from the Pakistani population.

## METHODS

This cross-sectional study was conducted at the section of Molecular Pathology Lab of Dow Diagnostic and Research Laboratory, Dow University of Health Sciences (DUHS) Karachi. Total 100 Blood samples were collected from diagnosed HIV drug-naive patients after filling out the consent form. Research proposal was approved by the ethical committee (IRB committee with reference no: IRB 906/DUHS/Approval/2017/102) of DUHS. A history was taken from all patients that were enrolled (supplementary file added for questionnaire). HIV viral RNA was extracted from plasma using the QIAamp Viral RNA Mini Kit, and Quantitative PCR (qPCR) was performed by Qiagen artus HI Virus-1 RG RT-PCR Kit.

DNA was extracted from the buffy coat using DNA Extraction kit (QIAamp). The beta-globin gene was amplified as an experimental control. Next the RT gene(*pol* region) was amplified by Nested PCR (Supplementary File) and PCR product was observed on agarose gel electrophoresis, then sent to Microgen Inc Korea for sequencing.

Sequences were trimmed and aligned with the reference sequences and CRFs of other subtypes of HIV-1 *pol* region available at Los Alamos HIV Database (HIV Databases. http://www.hiv.lanl.gov/content/index) using “Clustal-X” and “Mega 7.0” software for possible circulating subtype. Geographical linkages of HIV subtype sequences were aligned with sequences from different countries available on HIV (Los Alamos database) Databases.

From the aligned sequences, phylogenetic relationships and geographical linkages were determined using maximum likelihood (*ML*) with the Kimura model, and a bootstrap value of 1000 replicates with the help of MEGA 7.0 software.

Mutations in HIV-1 subgenomic sequences were analyzed by the HIV drug resistance database Stanford University (http://hivdb.stanford.edu/). Interpretation of drug resistance was made according to the Major and Minor mutation and resistance pattern (Supplementary File). For amino acid sequence variability analysis, sequences were translated using an expasy translate tool (https://web.expasy.org/translate/). Variability was evaluated by entering the protein sequence into the entropy one online tool available at the Los Alamos National Laboratory (LANL) HIV Sequence Database: www.hiv.lanl.gov/content/sequence/ENTROPY/entropy_one.html

## RESULTS

One hundred HIV-1 ELISA-positive patients were recruited of which, 2% were 1-10 years old, 6% were 11-20 years, 32% were 21-30 years old, 25% were 31-40 years old, 25% were 41-50 years old, 9% were 51-60 years old, and 1% were >60 years old. Gender distribution of patients revealed 84% were male and 16% were female. The patient’s marital status showed that 84% were married, whereas 16% were unmarried.

Out of positive patients, 70% were positive on Quantitative Real-Time PCR, and 30% were negative. RT region of *pol* gene amplification showed that 26% were positive, and only 16% were successfully sequenced for further analyses.

For genetic diversity analysis, a phylogenetic tree was reconstructed with 38 sequences of HIV-1 *pol* genes submitted to the HIV database from Karachi, and 88 reference sequences representing all the subtypes and CRFs. This study showed that 68.75% of sequences closely related to the reference subtype A1. 6.25% sequences closely related to the reference subtype B, CRF02_AG, CRF35_AD, CRF10_CD, and CRF11_cpx.

**Fig.1:**
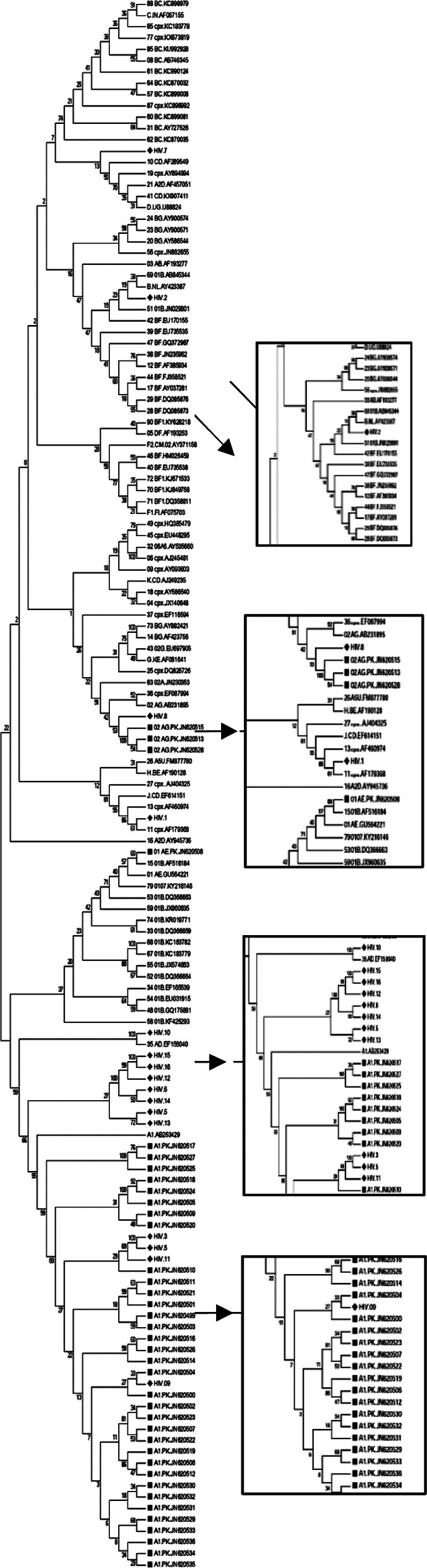
Phylogenetic tree constructed from HIV-1 using sequences from the current study, sequences reported from Karachi, and reference sequences. This tree represents the genetic diversity of HIV-1 by phylogenetic analysis; Red ♦ (Diamonds) represents our sequences, whereas green ▄ (box) represents the sequences from Karachi submitted to the HIV database. The tree was aligned with the reference sequences of countries.

Thirty eight sequences previously submitted from Karachi show that 89.76% sequences closely related to the reference subtype A1, 2.64% closely related to the reference subtype AE, and 7.38% closely related to the reference subtype CRF02_AG (Fig.1).

**Fig.2:**
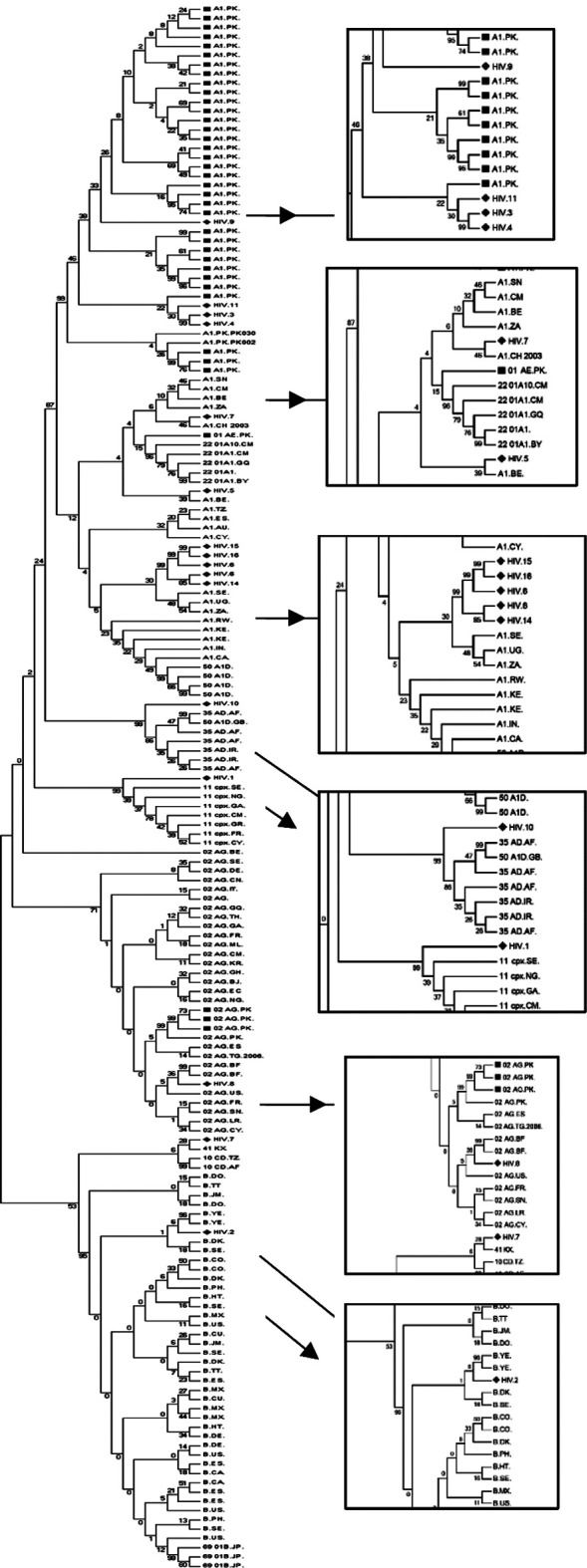
The phylogenetic tree was constructed from HIV-1 sequences from the current study, sequences reported from Karachi, and reference sequences of countries. This tree represents geographic linkages of HIV-1 using phylogenetic analysis; Red ♦ (Diamonds) representing our sequences, whereas green ▄ (box) represented the sequences from Karachi submitted to the HIV database. The tree was aligned with the reference sequences of countries that have circulating subtypes as in Karachi.

For possible geographic linkages of HIV-1 in Pakistan, 85 sequences representing 32 countries were downloaded from the database and used in the phylogenetic trees reconstruction, with the sequences from this study. Results showed that 31.25% of A1 sequences had close clusters with A1 sequences from Uganda, Sweden, and South Africa. 25% A1 showed a close cluster with previously reported A1 sequences from Pakistan. 6.25% A1 sequences closely clustered with the sequences of A1 from Belgium. 6.25% CRF35_AD sequence was crowding with a reference sequence of Afghanistan, 6.25% CRF11_cpx sequence was clustering with the reference sequence of Sweden, 6.25%, CRF02.AG sequence was clustering with the reference sequence of Burkina Faso. 6.25% CRF10_CD sequence was clustering with the reference sequence of Tanzania, and 6.25% B sequence was clustering with the reference sequence of Yemen (Fig.2).

Drug resistance was analyzed for NRTI drugs, including 3TC, ABC, AZT, FTC, TDF, and NNRTI drugs, including efavirenz EFV, ETR, NVP, and RPV. Results showed that many of these drugs were represented as H-LR, P-LLR, L-LR, and IR. Results from mutation analysis demonstrate that 50% of patients exhibit mutation whereas, out of mutated sequences, 12.5% showed mutation for NRTI drugs, and 62.5% were mutated for NNRTI drugs. It was also observed that 25% had a mutation for both NRTI and NNRTI drugs.

The most prevalent mutation for NRTI was M184V, observed in 75% of sequences. For NNRTI, it was E138A, which was observed in 80% of sequences. Additionally, 60% exhibited mutation in K103N, 40% showed V179D, 20% showed a major mutation for K13N, and 20% showed a major mutation for M230L. (Table-I)

**Table I:** Major mutations in the reverse transcriptase (RT) gene of HIV-1 in Pakistan.

		Major Mutation		Drug Resistance of RT								

S.No	Subptype	NRTI	NNRTI	3TC	ABC	AZT	FTC	TDF	ETR	NVP	RPV	EFV
1.	A1	NONE	E138A, V179D	S	S	S	S	S	L-LR	IR	IR	IR
2.	A1	NONE	E138A, V179D	S	S	S	S	S	L-LR	IR	IR	IR
3.	CRF50_A1D	M41L, M184V, T215Y	NONE	H-LR	IR	IR	H-LR	L-LR	S	S	S	S
4.	CRF50_A1D	NONE	NONE	S	S	S	S	S	S	S	S	S
5.	CRF 02AG	M184V	K103N	H-LR	L-LR	S	H-LR	S	S	H-LR	S	H-LR
6.	A1	NONE	E138A	S	S	S	S	S	PL-LR	S	L-LR	S
7.	CRF35_AD	M184V	K103N, M230L	H-LR	L-LR	S	H-LR	S	IR	H-LR	H-LR	H-LR
8	A1	NONE	K13N, E138A	S	S	S	S	S	PL-LR	H-LR	L-LR	H-LR
9.	CRF50_A1D	NONE	K103N	S	S	S	S	S	S	H-LR	S	H-LR

This table represents primary mutation and resistance pattern against NRTI and NNRTI drugs; S sensitive; L-LR low-level resistance; IR intermediate resistance; PL-LR Potential-Low Level resistance; H-LR High-Level resistance.

**Fig.3:**
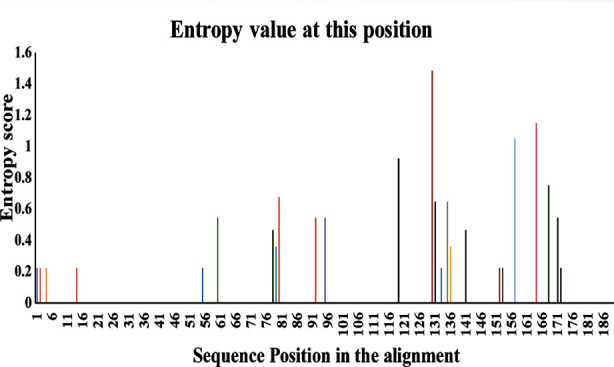
Drug Resistance of NRTI and NNRTI. X-axis represents drugs of NRTI as 3TC, ABS, AZT, FTC, TDF, and NNRTI as ETR, NVP, RPV, and EFV, whereas Y-axis represents no of patients in %.

In addition to major mutation, several minor mutations were observed for NRTI and NNRTI drugs. The most prevalent minor mutations analyzed were K122E, D177E, I293V, V35T, Q174K, and E291D. Drug resistance of NRTI drugs showed that 3TC showed H-LR to 18.75% of patients. FTC was H-LR to 18.75%, and TDF showed L-LR to 6.25% of patients (Fig.3). Simultaneously, ABC showed L-LR to 12.50% and IR to 6.25% of patients.

**Fig.4:**
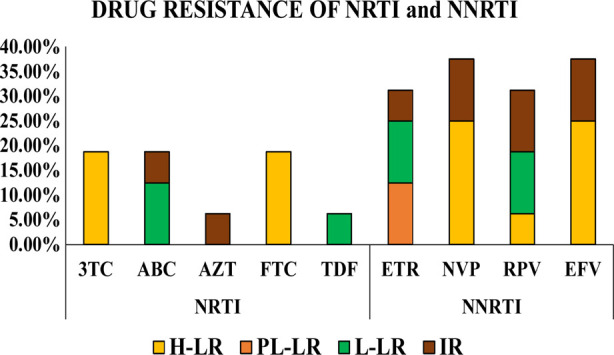
Analysis of genetic variability in amino acid sequence. To evaluate the variability in the RT gene, we carried out Shannon entropy analysis on patient-derived sequences. High peaks demonstrate that certain major RT resistance mutations occur more frequently.

Whereas drug resistance NNRTI drugs revealed that ETR showed P-LLR to 12.50%, L-LR to 12.50%, and L-LR to 6.25% patients. However, NVP showed H-LR to 25% and IR to 12.50% of patients. RPV showed H-LR to 6.25%, L-LR to 12.50%, and IR to 12.50% patients. EFV was showing H-LR to 25% of patients (Fig.3). Shannon entropy results revealed that certain regions of RT exhibited high sequence variability, especially at Amino Acids positions p.119, p.130, p.157, p.164 (Fig.4).

## DISCUSSION

The HIV epidemic is increasing globally, with many subtypes and CRFs emerging because of genetic diversity.^8^ Due to this, drug resistance mutations are also emerging. In Pakistan, the pattern of the HIV epidemic has changed from a low prevalence country to a concentrated epidemic.^9^ The country’s changing socioeconomic conditions, poor hygiene, an inappropriate healthcare system, cross-border migration, and high-risk activities may evolve the virus.^10^ Therefore, our study focused on investigating the genetic diversity, geographic linkages, and drug resistance pattern of HIV-1 circulating in this region.

Our results showed that subtype A is still the predominant subtype in Karachi, which was previously reported by Khan S *et al*.^11^ Our data also shows other subtypes emerging in the country, including B, C, CRFs. Furthermore, most of Pakistan’s sequences were subtype A.^11^

Since the first case of HIV was reported, other predominant HIV subtypes are slowly becoming prevalent in Pakistan.^12^ Our study reported that a few sequences were clustering with subtype B, similar to a recently published study that revealed 9.5% of sequences from the database were closely pressing with subtype B.^13^ Our study also reveals that 6.25% of sequences clustered with CRF02_AG. Chen Y *et al*. also submitted sequences in 2015 to the HIV database representing CRF02_AG,^14^ and Khan *et al*. in 2016 also submitted sequences representing the CRF02_AG.^15^ Link: www.hiv.lanl.gov/components/sequence/HIV/asearch/query_one.comp?se_id=KY445868).

CRFs like AE and AG were also reported from Pakistan, Khan et al. al. in 2016 submitted sequences that closely clustered with CRF01_AE.^15^ Link: www.hiv.lanl.gov/components/sequence/HIV/asearch/query_one.comp?se_id=KY445848. At the same time, another group reported that 17.10% of sequences clustered with HIV circulating recombinant form AE.^16^ As the recombination was observed, few new emerging subtypes are expected to be prevalent in the country. However, the scenario seems to be changing as previously in 2016, Chen Y *et al*. also reported new recombinant forms of HIV-1 in Pakistan CRF02/A1.^14^ Our results observed a few CRFs never before reported from Pakistan, like CRF11_CPX and CRF10_CD. According to the HIV database,^15^ 40% reported CRF circulating in the Central African Republic are CRF11_cxp. CRF35_AD is prevalent in Iran and Afghanistan, which share close borders with Pakistan, and people travel here for goods transportation and migration of refugees.^17^

These new strains may be introduced in the community due to the migration of HIV-positive individuals like laborers and sex workers from India, Iran, Afghanistan, the Middle Easterns, and African Countries.^13^ In 2017, the geographic linkage of the virus circulating in Pakistan showed that sequences submitted to the database till 2017 were closely clustered with sequences from Afghanistan, India, South Africa, Kenya, and Rwanda.^13^ Our results were similar, demonstrating that sequences were clustering with Afghanistan, African countries like Uganda and South Africa, and Middle Eastern countries like Yemen. However, 18.75% of sequences represent emerging subtypes and cluster with Afghanistan, Sweden, and Tanzania. These linkages of HIV circulating strains between countries might be due to infected individuals traveling to and from Pakistan without medical surveillance and medical clearance certificate.^13^ Therefore, laws regarding immigration policies should be revised to control further infectious transmission.

As the pattern of genetic diversity is changing, drug resistance is also increasing, and the virus is gaining High-Level Resistance from sensitivity against ART drugs. Sharaf *et al*. reported that 36.4% of patients showed resistance against NRTI drugs and point mutations, including M184V, D67G, K70R, with high prevalence.^7^ However, our data shows that 12.5% of patients were resistant to NRTI drugs. We observed several point mutations similar to those reported before,^7^ like M184V, in 75% of patients. 25% of patients showed point mutation for M41L. The discrepancy in results might be due to the difference in sample size and patients’ selection. A similar mutation, M184V was reported from Iran in 2015.^18^ Lin B *et al*. reported an identical mutation of M184V against NRTI drugs in the Chinese population. 19

Mutation against NNRTI drugs shows the greater impact of viruses becoming resistant.^20^ Sharaf *et al*. reported that 81.8% of patients have NNRTI mutations^7^ while sequences reported from Kuwait demonstrate 20% mutation against NNRTI drugs.^21^ These were similar to our results that demonstrated 62.5% NNRTI mutation. Among NNRTI drug-resistant mutations, the most prevalent point mutation was E138A, which was also previously reported.^7^ We observed that 80% of patients exhibited a point mutation of E138A and that 40% of patients have point mutation of K103N similar to that reported from Iran.^22^

According to the literature, a minor mutation has no drastic effect on drug resistance.^23^,^24^ However, if any major mutation is present and a minor mutation occurs, it causes an extreme change in resistance to RT.^24^ Shannon entropy analysis was performed to calculate the probability of upcoming major and minor mutations which demonstrated that on positions p.119, p.130, p.157, and p.164, the probability of attaining mutation is high. These positions have high genomic variation and may cause a major RT resistance mutation with a genomic variation on the same position.

### Limitations

Our study had few limitations, as more samples could be sequenced from different high- risk groups and sampling could be done from different cities of Pakistan, where there is a higher prevalence of HIV than Karachi, as well as cities with low prevalence should also be included.

## CONCLUSION

Our results reveal that subtype A is a predominant subtype in Karachi, whereas emerging subtypes B, CRF01_AE, CRF02_AG, CRF11_CPX, CRF35_AD, and CRF10_CD were also observed. Moreover, we report that geographically, the origin of the circulating virus represents that new subtypes were transmitted from neighboring, Middle Eastern, and African countries. Additionally, most patients showed major mutations with several minor mutations that tend to change the pattern of drug resistance from drugs being sensitive to achieving a High level of resistance. Therefore, ART is now causing High-Level resistance in patients with no therapeutic effect.

### Author’s Contribution:

**MZ:** Performed the experimental work & manuscript writing.

**MAQ:** Did the data analysis & manuscript editing.

**YR:** Did the sample, data collection and reviewed the manuscript.

**SK:** Conceived & designed the study & did bioinformatics analyses. He is also responsible for the integrity and accuracy of the study.
